# Impact of Smoking History on Response to Immunotherapy in Non-Small-Cell Lung Cancer: A Systematic Review and Meta-Analysis

**DOI:** 10.3389/fonc.2021.703143

**Published:** 2021-08-23

**Authors:** Wenhua Zhao, Wei Jiang, Huilin Wang, Jianbo He, Cuiyun Su, Qitao Yu

**Affiliations:** Department of Respiratory Oncology, Affiliated Tumor Hospital of Guangxi Medical University, Nanning, China

**Keywords:** immunotherapy, non-small cell lung cancer, smoking, clinical benefit, meta-analysis

## Abstract

**Objectives:**

To evaluate the impact of smoking history on the clinical benefit of immunotherapy in patients with non-small cell lung cancer (NSCLC).

**Methods:**

Twenty-three randomized clinical trials and seven real-world studies were included in this meta-analysis. Hazard ratios (HRs) and 95% confidence intervals (CIs) for overall survival (OS) and progression-free survival (PFS) and odds ratios for the overall response rate (ORR) were extracted. A fixed-effects or random-effects model was applied to obtain pooled estimates.

**Results:**

Data from 16 high-quality trials involving 10,643 NSCLC patients receiving either immunotherapy or chemotherapy/placebo enabled direct comparison of the survival impact of smoking. Anti-PD-1/PD-L1/CTLA-4 immunotherapy was found to significantly prolong OS and PFS as compared to chemotherapy/placebo in smokers (HR for OS, 0.76 [0.69–0.83], P<0.00001; HR for PFS, 0.65 [0.56–0.75], P<0.00001), and these trends were less or not significant in non-smokers (HR for OS, 0.91 [0.78–1.06], P=0.25; HR for PFS, 0.68 [0.45–1.03], P=0.07). Consistent results were obtained for the first-line or second/third-line use of immunotherapy and for non-squamous NSCLC patients only. Furthermore, the data from 7 trials and 7 real-world studies involving 4,777 patients receiving immunotherapy allowed direct comparison of therapeutic outcomes between smokers and non-smokers. Prolonged OS (HR 0.86 [0.75–0.99], P=0.04) and PFS (HR 0.69 [0.60–0.81], P<0.0001) and a higher response rate (ORR 1.20 [0.94–1.53], P=0.15) were observed in smokers compared to non-smokers receiving immunotherapy.

**Conclusions:**

Immunotherapy was found to have a greater benefit in NSCLC patients with a smoking history than in those who had never smoked.

## Introduction

Lung cancer remains a highly prevalent cancer and the leading cause of cancer-related death worldwide (1). Even with various therapeutic regimens available for NSCLC, the prognosis of many patients is still dismal. Continuous efforts have been made to improve outcomes in NSCLC patients, and some breakthroughs have been achieved in recent years, the most promising one being the development immunotherapy (2).

The basic approach of immunotherapy is to evoke an anti-tumor immune response by blocking an immune checkpoint, like programmed death-1/programmed death ligand-1 (PD-1/PD-L1) and cytotoxic T-cell lymphocyte antigen-4, and thereby achieving durable control of a tumor (3). Although remarkable improvements in clinical outcomes in multiple cancer types have been achieved by immunotherapy (4), only a limited percentage of NSCLC patients respond to the therapy, with less than 30% of NSCLC patients benefiting from immunotherapy (5). Thus, efficient markers to identify NSCLC patients who are most likely to respond to immunotherapy are urgently needed.

Several biomarkers, such as PD-1/PD-L1 expression on tumor cells, T-cell infiltration within the tumor microenvironment, and tumor mutation burden (TMB) (6–8), have been proposed to be associated with the therapeutic response of NSCLC to immunotherapy. However, the acquisition of these parameters requires tumor biopsy, which is not always possible, especially for patients with advanced disease. Thus, the ability to identify responders based on clinical characteristics would be of great clinical significance. Smoking history is a risk factor for pulmonary carcinogenesis, and smoking is known to alter the immune microenvironment and TMB in lung cancer, effects which have been linked with the therapeutic efficacy of immunotherapy (7–10). However, direct evidence for whether a smoking history is associated with the response to immunotherapy is lacking. Previous studies have hinted that NSCLC patients who are smokers might derive greater benefit from immunotherapy as compared to those who are non-smokers (11, 12). However, other reports have suggested that smoking history has no effect on the therapeutic outcome of immunotherapy (13, 14). The significance of smoking history in predicting the response to immunotherapy has yet to be systematically verified in a large-scale dataset.

Many clinical trials and real-world studies have provided data regarding immunotherapy-related outcomes and clinical characteristics like smoking history in NSCLC patients. However, these individual trials or studies have limited statistical power to validate a response difference related to smoking history. We thus carried out a meta-analysis by pooling the publicly available data to investigate the impact of smoking history on the therapeutic outcome of immunotherapy. We also evaluated the significance of smoking in different contexts (immunotherapy as first-line or not, adenocarcinoma or squamous cell carcinoma, etc.).

## Materials and Methods

### Literature Search Strategy and Study Selection

We carried out a systematic search of the PubMed, Embase, and Cochrane databases as well as conference websites including the World Conference on Lung Cancer (WCLC), American Society of Clinical Oncology (ASCO), and European Society for Medical Oncology (ESMO) websites for articles or abstracts with no language restriction or limitation on publication year (up to September 10, 2020). Relevant studies were searched using the following terms: “(lung neoplasms OR non-small cell lung cancer) AND (pembrolizumab OR Keytruda OR MK-3475 OR SCH 900475 OR nivolumab OR Opdivo OR BMS-936558 OR MDX-1106 OR ONO-4538 OR atezolizumab OR Tecentriq OR MPDL3280A OR RG7446 OR RO5541267 OR PD-1 OR PD- L1) AND trial”. Two authors independently carried out the searches. Studies were eligible only if they fulfilled the following inclusion criteria: (i) clinical trials or real-world studies; (ii) included NSCLC patients who received immunotherapy (anti-PD-1, anti-PD-L1, or anti-CTLA-4) (iii) reported hazard ratios (HRs, immunotherapy cohort *vs* control) for progression-free survival (PFS) and/or overall survival (OS) stratified by smoking history or reported clinical outcome data such as HRs or odds ratios (ORs) of clinical response for smokers *vs* non-smokers. Studies that failed to meet these inclusion criteria were excluded. We excluded studies if they contained participants who were also included in other studies.

### Data Extraction

The following information were extracted from the included studies: lead author, publication year, study categories (clinical trial or real-world study), study population, therapeutic regimens, line of treatment, histology (non-squamous v squamous), smoking status (never-smoker *vs* current or former smoker). For studies comparing clinical outcomes between an immunotherapy cohort (received immunotherapy only or in combination with chemotherapy) and a control cohort (received chemotherapy or placebo), the HRs for OS or PFS together with the 95% confidence intervals (CIs) were collected for smokers and non-smokers separately. For studies directly comparing clinical outcomes of immunotherapy (immunotherapy only or in combination with chemotherapy) between smokers and non-smokers, indexes of therapeutic efficacy including HRs for OS or PFS and the OR for the objective response rate (ORR) with the corresponding 95% CIs were collected for further pooled analysis.

### Statistical Analysis

All analyses were performed using Review Manager software (RevMan version 5.4; Oxford, UK). For studies containing data regarding the clinical benefits of immunotherapy *vs* chemotherapy/placebo, HRs (immunotherapy *vs* control) for smokers and non-smokers were pooled separately, which facilitated indirect comparisons of clinical benefits of immunotherapy between smokers and non-smokers. For studies containing data for direct comparison of the clinical benefits of immunotherapy in smokers *vs* non-smokers, HRs (smokers *vs* non-smokers) or ORs for ORR (smokers *vs* non-smokers) were accumulated to obtain pooled results for direct comparison. The χ^2^ test and I^2^ statistic were applied to assess statistical heterogeneity, with P<0.10 on the χ^2^ test and an I^2^ value >50% indicating the existence of heterogeneity. A random model was used to calculate the cumulative HRs, ORs, and their estimated 95% CIs when heterogeneity was observed among studies; otherwise, a fixed effect model was applied. Null hypothesis (the difference of immunotherapy effect between smokers and not smokers is zero) was tested by comparing the HR among with that of non-smokers following approaches: first, for each trial, an interaction trial-specific HR from the ratio of the reported HRs in smokers and non-smokers patients have to be calculated; second, these trial-specific interaction HRs across trials have to be combined using a random-effects model (15). For the pooled analysis of HRs (immunotherapy *vs* control) in smokers and non-smokers, subgroup analyses were performed for groups that received immunotherapy as first-line versus second/third-line treatment, as well as for the subgroup of non-squamous NSCLC patients. We did not perform a subgroup analysis for squamous NSCLC patients due to insufficient data. Publication bias was examined by funnel plot and Egger’s test. All CIs had two-sided probability coverage of 95%, and a P-value less than 0.05 was considered significant for all statistical tests.

## Results

### Characteristics of Included Studies

**Figure 1:**
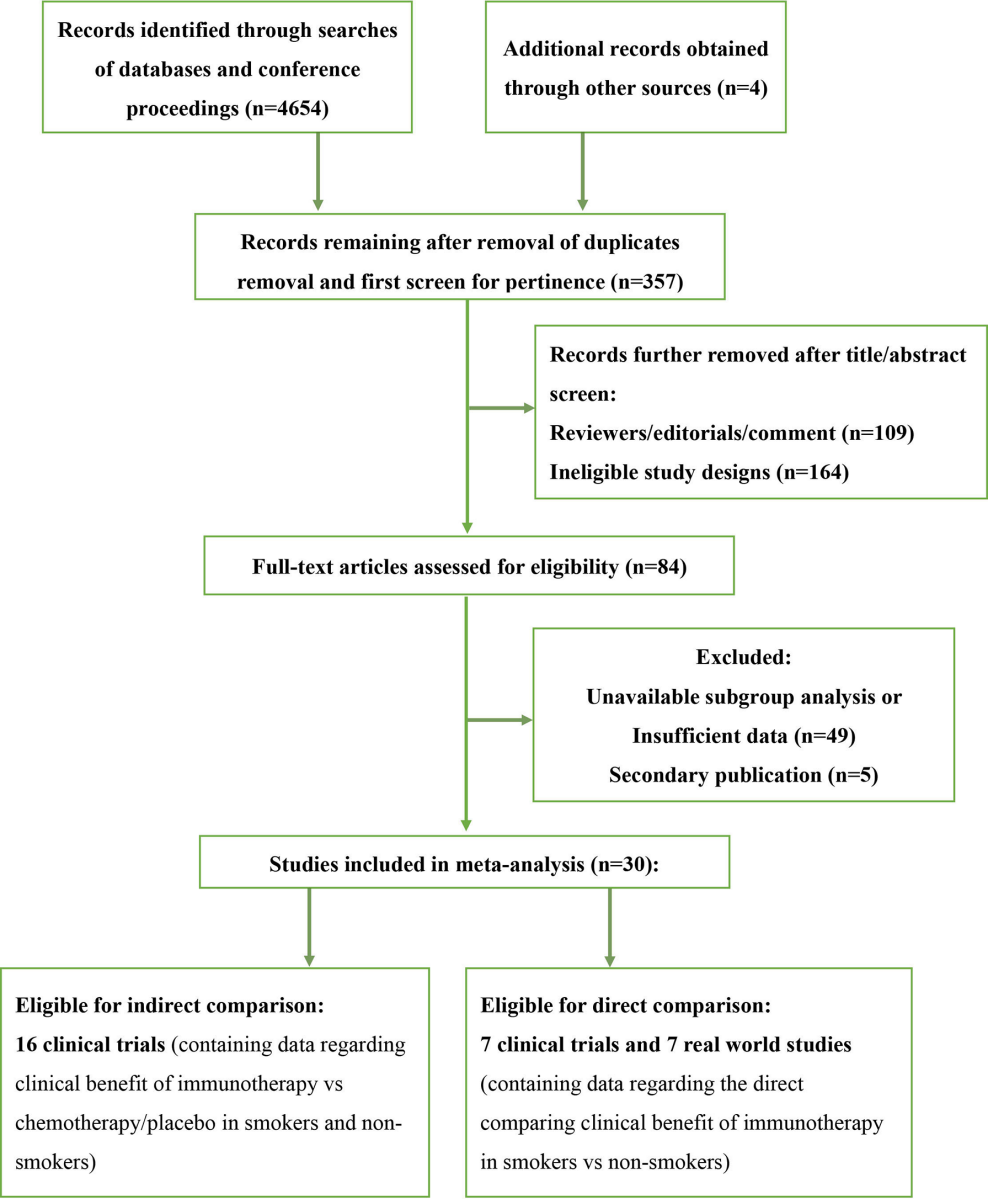
Flow chart of literature search strategy and study selection.

We screened 4,654 studies and identified a total of 23 clinical trials and 7 real-world studies that were eligible for inclusion in the present study (**Figure 1**). Sixteen phase-3 clinical trials involving 10,643 patients compared the observed clinical benefit between an immunotherapy cohort and a chemotherapy/placebo cohort and provided data for the smoking status of patients, and thus, facilitated an indirect comparison of the clinical benefit achieved in smokers versus non-smokers (**Table 1**). Patients in the immunotherapy cohort received immunotherapy as monotherapy, a combination of two types of immunotherapies, or immunotherapy combined with chemotherapy. The control cohort received chemotherapy as the control treatment in all of the clinical trials except one, in which placebo was administered. We also conducted a pooled analysis for direct comparison of the clinical benefit from immunotherapy between smokers and non-smokers with the data provided by 7 clinical trials and 7 real-world studies, which included 4,777 cases in total (**Table 2**). In the included studies, the most commonly applied immunotherapy was anti-PD-1 treatment, but some patients received anti-PD-L1 or anti-CTLA-4 therapy in selected studies. The basic information of all the included studies is summarized in **Tables 1**, **2**.

**Table 1:** Summary of the 16 clinical trials that provided data for indirect comparison.

Study	Trial type (name)	Population	Immuno therapy	Line of treatment	Study design	Sample size, n	Squamous, n	Non-squamous, n
Rittmeyer 2016 (16)	Phase 3 (OAK)	NSCLC	Anti-PD-1	2-3	Atezolizumab *vs* docetaxel	850	222	628
**Zhou 2019** (17)	Phase 3	Non-squamous	Anti-PD-1	1	Camrelizumab + chemo *vs* chemo	412	0	412
Carbone 2017 (18)	Phase 3 (CheckMate 026)	NSCLC	Anti-PD-1	1	Nivolumab *vs* platinum-based chemo	541	130	411
Barlesi 2018 (19)	Phase 3 (JAVELIN Lung 200)	NSCLC	Anti-PD-L1	2-3	Avelumab *vs* docetaxel	792	242	550
**Barlesi 2018-2** (20)	Phase 3 (IMpower132)	Non-squamous	Anti-PD-1	1	Atezolizumab + chemo *vs* chemo	578	0	578
Borghaei 2015 (21)	Phase 3 (CheckMate 057)	Non-squamous	Anti-PD-1	2-3	Nivolumab *vs* Docetaxel	582	0	582
West 2019 (22)	Phase 3 (IMpower130)	Non-squamous	Anti-PD-1	1	Atezolizumab + chemo *vs* chemo	723	0	723
Gandhi 2018 (23)	Phase 3 (KEYNOTE-189)	Non-squamous	Anti-PD-1	1	Pembrolizumab + chemo *vs* chemo	616	NA	592
**Paz-Ares 2019** (24)	Phase 3 (CheckMate 227 Part 2)	NSCLC	Anti-PD-1	1	Nivolumab + chemo *vs* chemo	755	212	543
Hellmann 2019 (25)	Phase 3 (CheckMate 227)	NSCLC	Anti-PD-1 + anti-CTLA-4	1	Nivolumab + ipilimumab *vs* chemo	1166	325	840
Reck 2016 (26)	Phase 3 (KEYNOTE-024)	NSCLC	Anti-PD-1	2-3	Pembrolizumab *vs* platinum-based chemo	305	56	249
**Reck 2020** (27)	Phase 3 (CheckMate-9LA)	NSCLC	Anti-PD-1 + anti-CTLA-4	1	Nivolumab + ipililumab + chemo *vs* chemo	719	62	138
Govindan 2017 (28)	Phase 3 (NCT01285609)	Squamous	Anti-CTLA-4	1	Ipilimumab +chemo *vs* chemo	749	749	0
Jotte 2020 (29)	Phase 3 (IMpower131)	Squamous	Anti-PD-1	1	Atezolizumab + chemo *vs* chemo	683	683	0
Antonia 2017 (30)	Phase 3 (PACIFIC)	NSCLC	Anti-PD-L1	1	Durvalumab *vs* placebo	713	326	387
Wu 2019 (31)	Phase 3 (CheckMate-078)	NSCLC	Anti-PD-1	2-3	Nivolumab *vs* Docetaxel	504	200	304

**Table 2:** Summary of the 7 clinical trials and 7 real-world studies eligible that provided data for direct comparison.

Studies	Trial/Real-world data	Population	Immuno-therapy	Treatment regimen	Line of treatment	Sample size, n	Squamous, n	Non-squamous, n
Gulley 2017 (13)	Phase 1b trial	NSCLC	Anti-PD-L1	avelumab	2+	184	53	114
Nishio 2016 (32)	Phase 2 trial (JapicCTI-132073)	Non-squamous NSCLC	Anti-PD-1	Nivolumab	2+	76	0	76
Nishio 2018 (14)	Phase 1b trial (KEYNOTE-025)	NSCLC	Anti-PD-1	Pembrolizumab	2+	38	6	29
Durm 2020 (33)	Phase 2 trial	NSCLC	Anti-PD-1	Pembrolizumab	2+	92	41	51
Goldberg 2020 (34)	Phase 2 trial	NSCLC	Anti-PD-1	pembrolizumab	1+	42	4	36
Feng 2017 (35)	Phase 2 trial	NSCLC	Anti-PD-1	Nivolumab	2+	648	293	354
Marina 2018 (36)	Phase 2 trial (ATLANTIC)	NSCLC	Anti-PD-L1	Durvalumab	3+	239	51	188
Kobayashi 2018 (37)	Real world data	NSCLC	Anti-PD-1	Nivolumab	2+	142	41	83
Lin 2018 (38)	Real-world data	NSCLC	Anti-PD-1	Nivolumab/pembrolizumab	1+	71	14	48
Khozin 2019 (39)	Real-world data	NSCLC	Anti-PD-1	Nivolumab/pembrolizumab	1+	1344	427	872
Barlesi 2020 (40)	Real-world data	NSCLC	Anti-PD-1	Nivolumab	1+	1420	982	438
Chen 2020 (41)	Real-world data	NSCLC	Anti-PD-1	Nivolumab/pembrolizumab	2	97	39	58
Morita 2020 (42)	Real-world data	NSCLC	Anti-PD-1	Nivolumab	1+	901	221	610
Weis 2019 (43)	Real-world data	NSCLC	Anti-PD-1/PD-L1	Nivolumab/atezolizumab	2	124	38	78

### OS or PFS Benefit From Immunotherapy in Smokers and Non-Smokers

**Figure 2:**
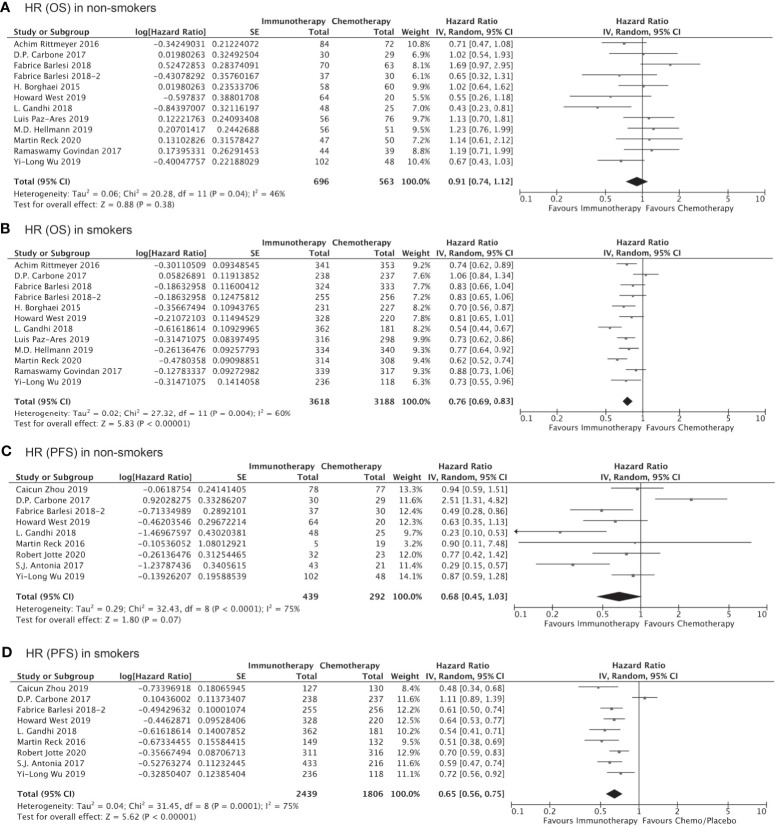
OS or PFS benefits of immunotherapy for NSCLC among smokers and non-smokers.

Of the 10,643 patients included in the 16 studies evaluating the clinical benefits of immunotherapy as compared to chemotherapy/placebo, 5,749 (54.01%) cases were randomized to the immunotherapy cohort. A total of 9,027 patients were identified as current smokers or former smokers, accounting for 82.36% of the study population. Compared to chemotherapy/placebo, immunotherapy significantly prolonged the OS of smokers (HR, 0.76; 95% CI, 0.69–0.83; P<0.001, **Figure 2B**) but demonstrated no significant OS benefit among non-smokers (HR, 0.91; 95% CI, 0.78–1.06; P=0.25, **Figure 2A**). With respect to PFS, the HRs were similar in both cohorts, although clinical significance was achieved in smokers (HR, 0.65; 95% CI, 0.56–0.75; P<0.001, **Figure 2D**) but not among non-smokers (HR, 0.68; 95% CI, 0.45–1.03; P=0.07, **Figure 2C**). Notably, the lack of statistical significance among non-smokers could be attributed to the smaller sample size, and the result does not necessarily prove that immunotherapy is less effective among non-smokers than smokers.

**Figure 3:**
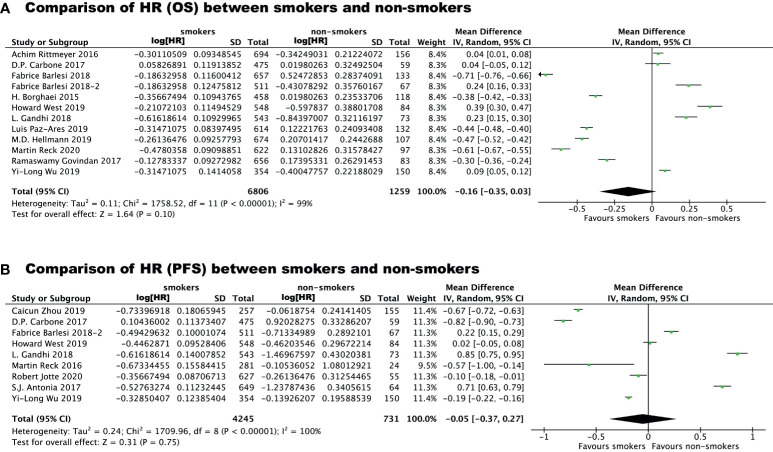
Comparison of Hazard Ratio regarding overall survival benefit **(A)** or progression free survival benefit **(B)** of immunotherapy *vs* chemo/placebo between smokers and non-smokers.

To avoid the risk of ecological bias, we further tested the difference in immunotherapy effectiveness between smokers and non-smokers by comparing the HRs for immunotherapy *vs* chemo/placebo in the two groups (**Figure 3**). We found that the HR for OS benefit from immunotherapy *vs* chemo/placebo in smokers was smaller than that in non-smokers, although the difference was not statistically significant (P=0.10), which could be attributed to high heterogeneity among studies (I^2 =^ 99%). The HR for PFS benefit from immunotherapy *vs* chemo/placebo among smokers was similar to that in non-smokers (mean difference: -0.05 [-0.37, 0.27], P=0.31, I^2 =^ 100%).

### Subgroup Analyses

**Figure 4:**
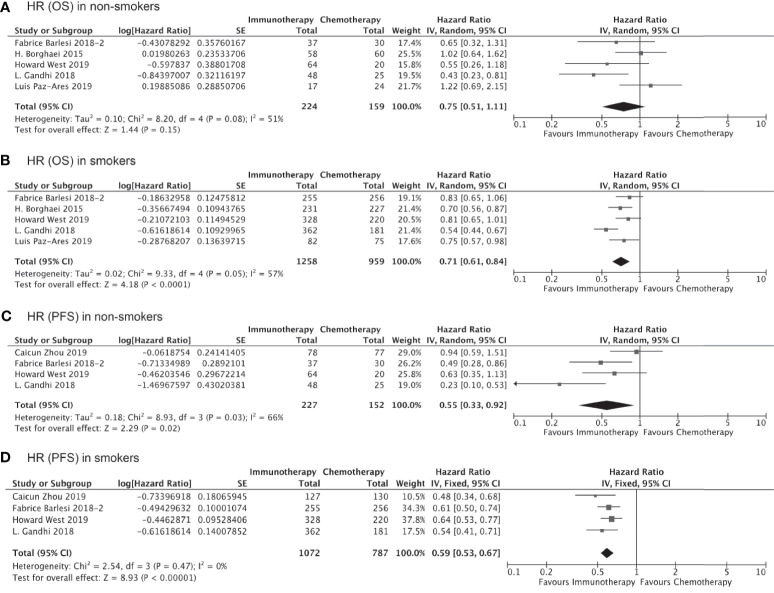
OS or PFS benefits from immunotherapy for non-squamous NSCLC among smokers and non-smokers.

We also carried out subgroup analyses indirectly comparing outcomes in subgroups with specific histological subtypes or receiving immunotherapy as first- versus second/third-line treatment. A total six trials containing data specific to non-squamous lung cancer were pooled to evaluated the impact of smoking on the clinical benefit of immunotherapy *vs* chemotherapy for these patients. As shown in **Figure 4**, smokers with non-squamous lung cancer experienced significantly prolonged OS (HR, 0.71; 95% CI, 0.61–0.84; P<0.0001, **Figure 4B**) and PFS (HR, 0.59; 95% CI, 0.53–0.67; P<0.0001, **Figure 4D**) after receiving immunotherapy as compared to chemotherapy. Immunotherapy could also significantly improve PFS in non-smokers with non-squamous lung cancer (HR, 0.55; 95% CI, 0.33–0.92; P=0.02, **Figure 4C**), although the improvement in OS was not significant (HR, 0.68; 95% CI, 0.45–1.03; P=0.15, **Figure 4A**). We did not carry out this subgroup analysis for patients with squamous cell lung cancer due to the insufficient number of studies available.

**Figure 5:**
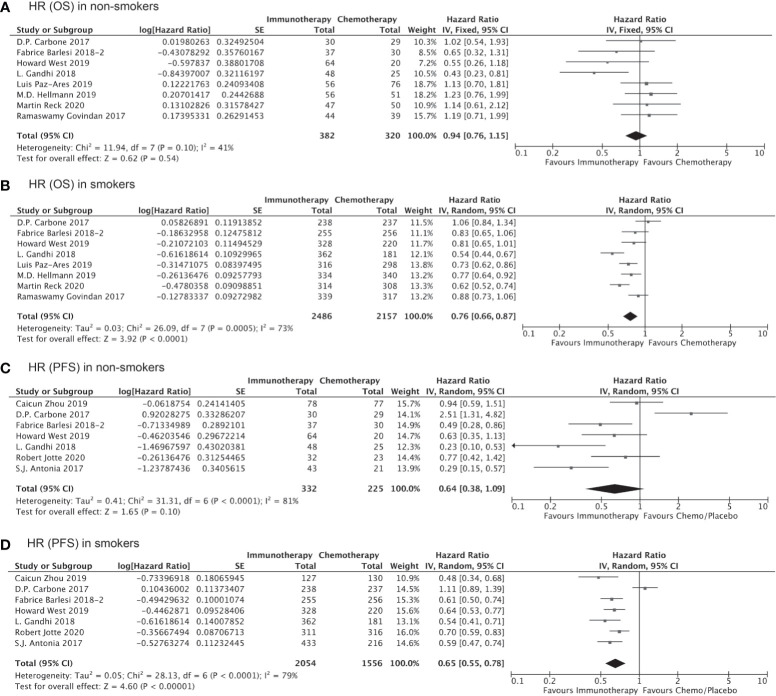
OS or PFS benefits from immunotherapy as first-line treatment among smokers and non-smokers.

**Figure 6:**
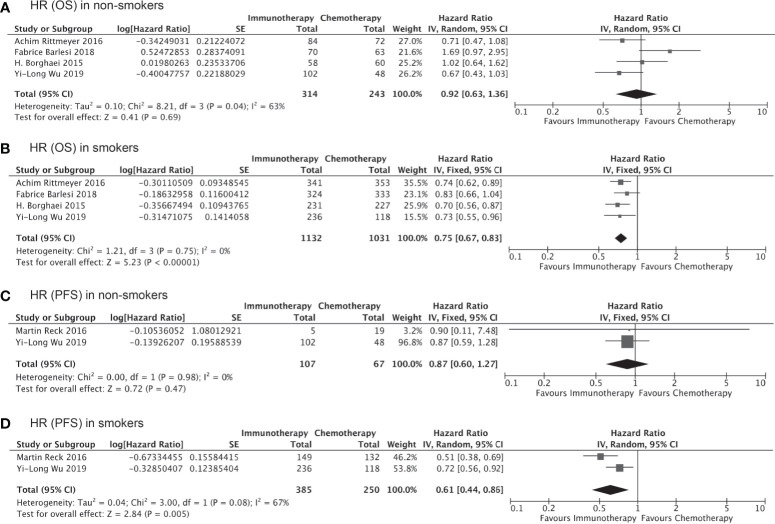
OS or PFS benefits from immunotherapy as second- or third-line treatment among smokers and non-smokers.

Of all the clinical trials included in our indirect comparison, 11 trials administered immunotherapy (alone or as combination therapy) as the first-line treatment, whereas the remaining 5 studies administered immunotherapy or chemotherapy as second- or third-line treatment. Pooled analysis of the data from these 11 trials involving 7,610 treatment-naïve patients showed that the OS (HR, 0.76; 95% CI, 0.66–0.87; P<0.0001, **Figure 5B**) and PFS (HR, 0.65; 95% CI, 0.55–0.78; P<0.00001, **Figure 5D**) were significantly prolonged among smokers who received immunotherapy as first-line treatment compared to those received chemotherapy/placebo as the first-line treatment, whereas no significant improvements in OS (HR, 0.94; 95% CI, 0.76–1.15; P=0.54, **Figure 5A**) and PFS (HR, 0.64; 95% CI, 0.38–1.09; P=0.10, **Figure 5C**) with immunotherapy were observed for non-smokers. Similarly, the clinical benefits of immunotherapy versus chemotherapy as second- or third-line treatment in terms of prolonged OS and PFS were significant only among smokers (HR for OS, 0.75 [0.67–0.83], P<0.00001, **Figure 6B**; HR for PFS, 0.61 [0.44–0.86]; P=0.005, **Figure 6D**) and not among non-smokers (HR for OS, 0.92 [0.63–1.36], P=0.69, **Figure 6A**; HR for PFS, 0.87 [0.60–1.27]; P=0.47, **Figure 6C**). Importantly though, the insignificant results for non-smokers may be due to the small sample size of this group.

### Direct Comparison of Therapeutic Outcomes Between Smokers and Non-Smokers

**Figure 7:**
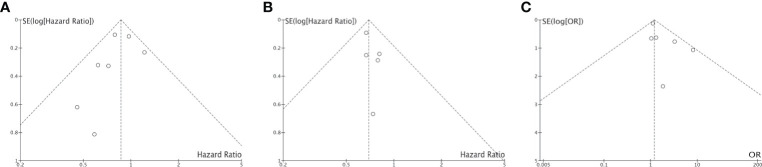
Funnel plot for publication bias of the direct comparison of HR-OS **(A)**, HR-PFS **(B)** and ORR **(C)** between non-smokers and **(C)** smokers.

**Figure 8:**
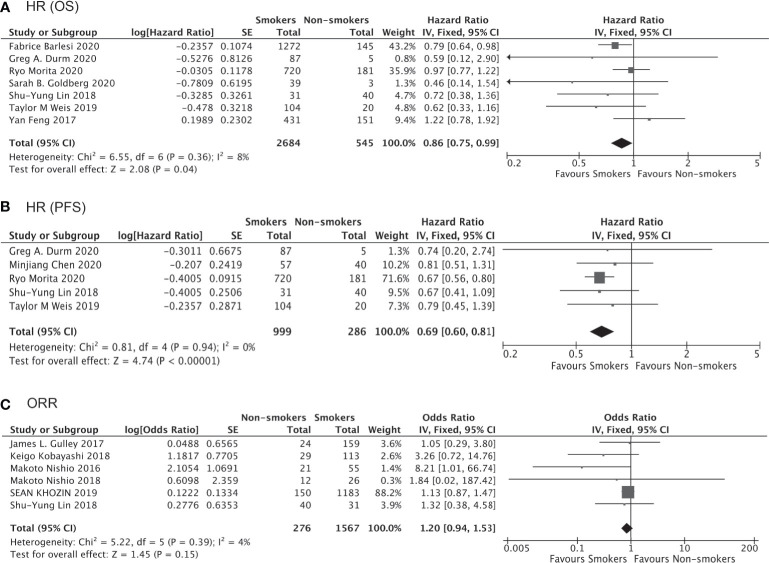
Direct comparison of immunotherapy outcomes between smokers and non-smokers with NSCLC.

Of the 4,777 patients included in 9 clinical trials and 7 real-world studies that directly compared the clinical outcomes of anti-PD-1/PD-L1 immunotherapy (monotherapy or combined with chemotherapy) between smoker and non-smokers with NSCLC, 3,098 (64.85%) patients were current or former smokers. There was no observed publication bias according to Egger’s test (**Figure 7**). The pooled analysis demonstrated that smokers achieved better OS (HR, 0.86; 95% CI, 0.75–0.99; P=0.04, **Figure 8A**) and PFS (HR, 0.69; 95% CI, 0.60–0.81; P<0.00001, **Figure 8B**) compared with non-smokers after receiving immunotherapy. Smokers also tended to achieve a higher ORR than non-smokers, although the difference was not statistically significant in the pooled analysis (OR, 1.20; 95% CI, 0.94–1.53; P=0.15, **Figure 8C**). We did not perform subgroup analyses for direct comparisons due to insufficient data availability.

## Discussion

As a well-known risk factor for lung cancer, smoking history plays a role in oncogenesis by altering tumor cells directly or by influencing the tumor microenvironment (44, 45). However, the potential effect of smoking history on the clinical outcomes of immunotherapy remains to be confirmed. This meta-analysis in which all the available data from clinical trials and real-world studies were pooled provides a comprehensive and systematic review of the impact of smoking history on the therapeutic outcomes of immunotherapy in NSCLC patients. We found that the survival benefit of immunotherapy over chemotherapy/placebo was more significant among smokers than among non-smokers, independent of the histologic subtype or line of treatment. Also, patients with a smoking history benefited more from immunotherapy than did non-smokers. The present meta-analysis enhances the evidence that smoking history, as a clinical parameter, can be applied as a predictor of immunotherapy efficacy and guide patient’s selection for immunotherapy among NSCLC patients.

Since the ground-breaking discovery of checkpoint blockade through anti-PD-1– and anti-PD-L1–based immunotherapy and the successful application of these immunotherapies in lung cancer, melanoma, etc., continuous efforts have focused on methods for identifying the patients most likely to respond to immunotherapy. Even though lung cancer is one of the cancer types that respond best to immunotherapy, only 20~30% of patients have been found to actually benefit from immunotherapy (5). PD-1 and PD-L1, as the direct targets of checkpoint blockade, have been widely applied as biomarkers in attempts to predict the potential response to immunotherapy (38). However, accumulating evidence indicates that the expression status of PD-1 or PD-L1 alone is insufficient to determine which patients should be offered PD‐1– or PD‐L1–targeting therapy (46). A tremendous amount of work has focused on the development of complicated predictive algorithms integrating genomic and tumor microenvironment features that can identify patients who are likely to benefit from immunotherapy (6–8), but little attention has been paid to clinical parameters that might also facilitate responder identification. Several studies have aimed to evaluate the significance of smoking history on the clinical outcomes of immunotherapy, with inconsistent findings and some studies reporting no significant relevance, which in no small part, can be attributed to the modest sample sizes of the study populations (7–10). Pooled analyses of the previously available data involving a large number of patients can provide much more solid conclusions regarding this issue. A review of the literature published through 2019 investigated the role of tobacco smoking in immunotherapy targeting PD-1/PD-L1 by summarizing the findings of clinical trials and reached the conclusion that NSCLC in current/former smokers responded better than that in non-smokers to immunotherapies (11). However, the conclusion was derived from a descriptive summary of previous findings, and no statistical evidence was derived from pooled analyses. Also, only a limited number of studies were included in that previous review, which restricted the feasibility of pooled analyses. With increasing numbers of relevant clinical trials as well as real-world studies published recent years, the now abundant data facilitated the pooled analyses in the current study. We evaluated the impact of smoking history on the outcomes of immunotherapy by demonstrating the survival gains with immunotherapy versus chemotherapy/placebo in smokers and non-smokers separately and also by directly comparing the clinical outcomes of immunotherapy between smokers and non-smokers. All of the different analyses in our study support the conclusion that immunotherapy offers better survival benefits in smokers compared with non-smokers. Another recently published study also performed meta-analysis to evaluate the impact of smoking on the therapeutic outcome of immunotherapy (12). Although the conclusions are similar, our study still outperforms the published one in terms of: a much larger sample size with several newly published clinical trials included; the impact of smoking status on the therapeutic efficacy of immunotherapy was evaluated not only by indirect comparison (evaluating the clinical benefit of immunotherapy in smokers and non-smokers separately), but also by direct comparison (comparing the therapeutic outcome of smokers with that of non-smokers after immunotherapy); systematic subgroup analysis was carried out to preclude potential confounding effects.

Importantly, although the survival improvement achieved with immunotherapy among non-smokers was not as significant as that among smokers, we cannot conclude that smokers benefit more from immunotherapy than non-smokers based only on the results of this analysis. Because the sample size of non-smokers analyzed in our study was far smaller than that of smokers, the statistical insignificance in non-smokers could be attributed to the small sample size. To better assess the differences in pooled OS-HRs and PFS-HRs for smokers and non-smokers, we further carried out an interaction test, which showed a relatively improved HR for smokers but without statistical significance. The statistical insignificance in these results could also be attributed to the high heterogeneity among studies included. Overall, the results derived from indirectly comparing the immunotherapy benefit between smokers and non-smokers cannot provide solid evidence that immunotherapy works better for smokers. Results derived from a direct comparison will better support the conclusion that smokers may benefit more from immunotherapy as compared to non-smokers.

The next logical question is why does a smoking history contribute to a better response to immunotherapy. The first and most obvious explanation is the correlation between smoking and TMB (47). TMB is another widely accepted biomarker for predicting the immunotherapy response among patients (48). It is widely acknowledged that NSCLC and melanoma are the two cancer types that most benefit from immunotherapy, and this has largely been attributed to the high TBM in both cancer types (49). While ultraviolet exposure is the major cause of DNA damage and elevated TMB in melanoma as a skin cancer, tobacco exposure likely contributes to the high TMB in lung cancer. A previous study showed that lung cancers in smokers had a significantly higher TMB compared with lung cancers in never-smokers (49). A high TMB contributes to the production of a higher abundance of neoantigens, which facilitates the recognition of cancerous cells by the immune system. Also, accumulation of neoantigens on the surface of tumor cells can stimulate the recruitment of cytotoxic immune cells into the tumor microenvironment, which will further boost the therapeutic efficacy of immunotherapy.

Additionally, smoking may exert an impact on the tumor microenvironment in a manner beyond TMB. In some inflammatory pulmonary disorders, such as emphysema and chronic obstructive pulmonary disease, smoking is thought to play a role in skewing the local immune microenvironment to a proinflammatory phenotype (50). Previous studies have also reported that the immunologic homeostasis within the tumor microenvironment is less compromised in never-smokers compared with ever-smokers (51, 52). It is believed that immune cells are recruited in response to tobacco exposure, in an attempt to minimize the damage induced by the carcinogenic substance *via* a pro-inflammatory reaction (53). However, the immune cells could also partially contribute to the harmful tumor microenvironment that promotes tumor growth (54). Smoking can influence the tumor microenvironment not only during the stage of tumor initiation, but may continue its effect throughout the process of tumor progression. For example, tobacco exposure was reported to polarize macrophages to a proinflammatory phenotype, M1 (55). Macrophages, as the major component within the tumor microenvironment, dictate the recruitment of other immune cells based on their functional status, with the immune suppressive macrophage phenotype suppressing T-cell infiltration and the proinflammatory macrophage phenotype efficiently promoting immune cell recruitment (56). Immune cell infiltration, and specifically infiltration of T cells, is believed to be a perquisite for immunotherapy to exert a therapeutic effect (57). As a substitute for direct examination of tumor tissue obtained by biopsy, smoking history might also convey implicit information regarding the status of the tumor immune microenvironment.

Smoking status was long ago found to correlate with certain oncogene mutations in lung cancer, with mutation of oncogenes such as *KRAS* commonly found in smokers (58). On the other hand, mutations of *EGFR*, *ROS1*, and *EML4-ALK* fusion occur more frequently in non-smokers (51). Accumulating studies have proven that lung cancers harboring oncogenic alterations in *ROS1* or *EML4-ALK* respond poorly to immunotherapy (59), whereas KRAS mutations are associated with an improved response to immunotherapy (60). Differences in the oncogene mutation spectrum may contribute to the differences in therapeutic outcome of immunotherapy among smokers and non-smokers. Furthermore, previous research has suggested that NSCLC in smokers demonstrates significantly higher expression of PD-L1 (61), which could also contribute to the predictive value of smoking for the response to immunotherapy.

Even with the comprehensive nature of our findings, the limitations of the present study need to be considered. First, as a meta-analysis carried out by pooling the published data, analysis of data from individual patients was not possible in our study. Thus, we could not control the test cohort and control cohort to make then comparable across all the studies. Differences in study design, therapeutic dosages, tumor stages, and patients’ performance status could be potential confounding factors. Secondly, significant heterogeneity among the included studies was observed in the analysis of OS and especially PFS. Heterogeneity among studies might be attributed to differences in study design, therapeutic regimens, tumor stages, follow-up system and the definition of disease progression across different study centers. Thirdly, we did not carry out subgroup analyses according to tumor stage, oncogenic alteration (e.g., *EGFR* mutation, *EML4-ALK* fusion, *KRAS* mutation), expression status of PD-1/PD-L1, etc. due to the limitations of the data. Last but not least, the study design of real-world studies may have been less well-organized as compared to that of clinical trials, which would compromise the quality of the reported data and thus might compromise the evidence level.

## Conclusions

In conclusion, our study systematically evaluated the impact of smoking history on the therapeutic response to immunotherapy in terms of survival benefit and ORR. We demonstrated that patients with a smoking history achieved longer survival times than non-smokers after receiving immunotherapy, through comparisons of the survival gains with immunotherapy versus chemotherapy/placebo between smokers and non-smokers, and by pooling the direct comparisons of survival gain and response rate with immunotherapy between smokers and non-smokers. Smoking history could be a simple index that guide the selection of potential responders to immunotherapy among NSCLC patients.

## Data Availability Statement

The raw data supporting the conclusions of this article will be made available by the authors, without undue reservation.

## Author Contributions

WZ, WJ, HW, and JH conceived and designed research. WZ, WJ, and QY collected data and conducted research. WZ, WJ, and CS analyzed and interpreted data. WZ and WJ wrote the initial paper. HW and JH revised the paper. QY had primary responsibility for final content. All authors contributed to the article and approved the submitted version.

## Funding

The present study was supported by grants from the Guangxi Medical and Health Appropriate Technology Development and Promotion Application Project (grant no. S2019049), Clinical Research Specific Funding Project of Wu Jieping Medical Foundation (grant no. 320.6750.19088-65) and Medical Research Funding Project of Beijing Medical and Health Public Welfare Foundation (grant no. TA201901).

## Conflict of Interest

The authors declare that the research was conducted in the absence of any commercial or financial relationships that could be construed as a potential conflict of interest.

## Publisher’s Note

All claims expressed in this article are solely those of the authors and do not necessarily represent those of their affiliated organizations, or those of the publisher, the editors and the reviewers. Any product that may be evaluated in this article, or claim that may be made by its manufacturer, is not guaranteed or endorsed by the publisher.

## Abbreviations

NSCLC, Non-small-cell Lung Cancer; ICI, Immune checkpoint inhibitor; PD-1, Programmed death-1; PD-L1, Programmed death ligand-1; CTLA-4, Cytotoxic T-lymphocyte-associated protein 4; OS, Overall survival; PFS, Progression-free survival; ICs, Confidence intervals; ORR, Overall response rate; HR, Hazard ratios; TMB, Tumor mutation burden.
